# Development and validation of a multiplex 19 X-chromosomal short tandem repeats typing system for forensic purposes

**DOI:** 10.1038/s41598-020-80414-x

**Published:** 2021-01-12

**Authors:** Juan Jia, Xu Liu, Qingwei Fan, Chen Fang, Mengchun Wang, Jiarong Zhang, Wanting Li, Linyu Shi, Xiaomeng Zhang, Chuguang Chen, Zailiang Yu, Chen Li, Keming Yun, Jiangwei Yan

**Affiliations:** 1 School of Forensic Medicine, Shanxi Medical University, Taiyuan, 030001 China; 2 Beijing Center for Physical and Chemical Analysis, Beijing, 10089 China; 3 Beijing Engineering Technology Research Centre of Gene Sequencing and Gene Function Analysis, Beijing, 100089 China; 4 Beijing Microread Genetics Co., Ltd, Beijing, 100044 China

**Keywords:** Genetic markers, Genotype

## Abstract

X-chromosome short tandem repeat (X-STR) markers are a powerful complementary system used for paternity and forensic casework. This study presents the development and validation of a new highly efficient multiplex-fluorescent-labeled 19 X-STR typing system, including DXS10079, DXS101, DXS10135, DXS10162, DXS6795, DXS6800, DXS6803, DXS6807, DXS6809, DXS6810, DXS7133, DXS7423, DXS981, DXS9902, DXS9907, GATA165B12, GATA172D05, GATA31E08 and HPRTB along with sex-typing locus, amelogenin. The system was validated according to guidelines issued by the Scientific Working Group on DNA Analysis Methods. Allele frequency and forensic parameters were investigated from 1085 (494 males and 591 females) unrelated Beijing Han individuals, the combined power of discrimination by the 19 X-STR loci in females and males, as well as the combined mean exclusion chance in trios and duos, were 0.999999999999999995, 0.99999999995, 0.9999999995, and 0.9999996, respectively. The results demonstrate that this multiplex system is robust and reliable, and considered to be a powerful tool for forensic application.

## Introduction

Short tandem repeats (STRs) are well-established and widely applied in the field of forensic science for individual identifications and paternity testing^1^. STRs loci are not only confined to the autosomal chromosomes but also presented on the X and the Y chromosome. In males, the X chromosome can therefore be considered as a haploid genome similar to the Y chromosome. Two females share the same biological father if they also share an X chromosome. Therefore, X-chromosomal STRs (X-STRs) can be used as a complementary tool in complex kinship analysis^2^ and deficiency cases^3,4^. These authors contributed equally to this work.

In this study, we developed a new highly efficient multiplex 5 fluorescent dye labeled 19 X-STRs typing system. The multiplex typing system was validated for robustness, sensitivity, precision, sizing accuracy, species specificity, performance against DNA mixtures, and stutter according to Scientific Working Group on DNA Analysis Methods (SWGDAM) guidelines (http://www.fbi.gov/hq/lab/fsc/backissu/july2004/standards/2004_03_standards02.htm). Allele frequency and forensic parameters were investigated from 1085 (494 males and 591 females) unrelated Beijing Han individuals. We believe that these data and the present multiplex system will prove useful in future human population genetic and forensic studies.

## Materials and methods

### Samples and DNA extraction

Blood samples were collected on FTA Cards (GE Healthcare/Whatman) from 1085 (494 males and 591 females) healthy unrelated individuals who provided informed consent. FTA cards were punched using a 1.2 mm BSD600-DUET stiletto instrument (BSD, Australia), and samples were placed in 96-well plates. DNA was extracted using Chelex-100^9^ , and quantified on a 7500 Real-time PCR System (Life Technologies, CA, USA) using Quantifiler Human DNA Quantification Kit (Life Technologies, CA, USA). Control DNA samples 9947A and 9948 were purchased from Promega Corporation (WI, USA), and Control DNA F312 was obtained from Microread Genetics Incorporation (Suzhou, China).

Non-human DNA samples were collected from chicken, mouse, cat, dog, pig, sheep, donkey, and soil microorganisms to evaluate species specificity. Those samples were donated from Animal Zoo (Taiyuan, China) and the Laboratory Animal Center of Shanxi Medical University. Whole blood samples were collected from chicken, mouse, cat, dog, pig, sheep, donkey and DNA was extracted using QIAamp Blood Kit (Qiagen, Hilden, Germany). Soil sample was collected and soil microorganisms DNA was extracted using Soil Microbial Genomic DNA Extraction Kit (Tiangen Biotech, Beijing, China). The DNA concentration in non-human samples was determined by absorbance at 260 nm on a UV-2802S UV/Vis Spectrophotometer (UNICO, Shanghai, China).

The project and informed consent were approved by the Ethical Committee of Shanxi Medical University (Issued Number: 2019s110224) and the ethical principles of the 2000 Helsinki Declaration of the World Medical Association (http://www.uma.net/e/policy/b3.htm) were followed.

### Locus selection and characterization

Nineteen X-STRs (DXS10079, DXS10135, DXS7423, HPRTB, DXS6809, DXS7133, DXS7423, DXS9902, GATA172D05, GATA31E08, DXS101, DXS10162, DXS6795, DXS6800, DXS6803, DXS6807, DXS6810, DXS981, DXS9907, and GATA165B12) and amelogenin were selected for the new multiplex typing system (Fig. S1). STR loci were selected as follow considerations: (1) covered the whole X chromosome; (2) the lowest physical distance between two loci was above 5 Mb; (3) no any two loci were located at the same linkage group of the 4 known linkage groups (http://www.chrx-str.org); (4) high heterozygosity (≥ 0.4); (5) suitable for multiplex PCR primer designing^1^. Chromosomal location, physical position, genetic distance, repeat motif, and observed allele range at each locus are listed in Table 1.

**Table 1:** Loci and primer characteristics.

Locus	Genbank (UniSTS)	Chromosome location	Physical distance (Mb)	Genetic distance (cM)	Motif	Allele range	Primer sequence (5′-3′)	Tm (℃)	Amplicon size(bp)	Dye label
DXS6795	G09877	p22.11	23.254	44.24	AAT	9–14	F-CATCCATCCCCTAAACCTCTCATG	60.28	118–155	FAM
							R-TTTCAGCTCCTAGGTGACTGGGA	56.62		
DXS6803	G08107 (41,731)	q21.2	86.318	99.4	TCTA	10–14.3	F-GAACATATGCTACTTTATCTCGA	56.7	177–218	
							R-GCATATAAATTTTGAATTAAAG	57.18		
DXS6807	G09662 (44,289)	p22.33	4.753	14.76	GATA	11–16	F-TATGAAAAGAAAGGAAGCACCAA	52.67	240–277	
							R-ACAGGAGATATATAGTAAAAATAC	57.83		
DXS9907	G27255 (39,433)	p21.1	32.01	55.32	CTAT	9–15	F-TTCTCCCATCTCAGCCTCCTG	54.74	291–337	
							R-ACAAAGGATGTTAAAGCATCATC	54.43		
DXS7423	95,583	q28	149.46	184.19	TCCA	14–17	F-CACCCAGATTTCCTCCCCA	54.54	347–395	
							R-GCCACTGGGGACATGTGAATGG	57.88		
AMEL	AC002366.1 (AC013412.3)	X: p22.1–22.3 Y:p11.2			N.A	X, Y	F-CCCTGGGCTCTGTAAAGAATA	56.8	99–107	HEX
							R-ATCAGAGCTTAAACTGGGAAGCTG	56.97		
GATA172D05	498,601	q23	113.061	124.36	TAGA	6–12	F-TACTGTTTAACCAGTACAAAGTT	51.99	124–168	
							R-ATAGATATAGGTATTGATATAG	54.5		
DXS101	98,951	q22.1	101.3	116.15	CTT/ATT	20–27	F-ACAAAGATAATACACACATATTCAT	57.75	179–231	
							R-AGTGAGCATACATACATGTATCT	57.74		
DXS9902	G27261(73,076)	p22.2	15.234	32.32	GATA	7–14	F-AATGGAGTCTCTGGGTGAAGAG	56.52	244–288	
							R-CATATAATTATTTTCTTTG	56.65		
DXS7133	G08113(50,709)	q22.3	108.928	118.18	ATAG	6–12	F-ATATCTCCACTTCCAAAAGGGG	58.65	320–351	
							R-CTGTTCACCTTTCCGAGTCTTC	59.67		
DXS6810	G09983(7699)	p11.3	42.804	75.12	CTAT	16–20	F-ACCTCAGGTGATCCACCTGCCTCG	56.16	365–395	
							R-CATAGCTTAGCAAACAGCTGTATG	53.95		
GATA31E08	G08104(15,349)	q27.1	140.062	160.54	AGGG/AGAT	6–13	F-TGTTTATCATACTAGATATAGATA	54.7	107–145	TAMRA
							R-AGATACATGTATGTATGCTCACT	58.51		
DXS6800	G09609(58,372)	q13.3	78.567	97.49	TAGA	15–22	F-TGAAATATTGGGGGCTGGTTCC	55.1	165–207	
							R-CTTGCTCCTCAGCTTGCAGAGGG	55.18		
DXS981	99,675	q13.1	68.114	92.81	TATC	12–16	F-AAGTCACCACCATATTGTTCCTTGA	52.09	213–249	
							R-GGAAAAGAAGTAGACATACTTC	54.02		
DXS10162	NC_000023	q11.1	61.8	90.65	TCTT	15–21	F-TCACTAAGCAGTTTCATAGATAG	63.49	275–345	
							R-GGAAAACCAGGTTATCCCCAG	60.26		
DXS6809	G08112(69,546)	q21.33	94.825	108.12	ATCT/CTAT	29–36	F-TCTGGAGAATCCAATTTTGC	53.4	347–404	
							R-GGCCTCACTTAATGGATGAAGCA	54.78		
GATA165B12	G10699(63,387)	q25	120.706	136.18	AGAT	8–12	F-CTGATATACGGATAGATGATTG	57.25	86–128	ROX
							R-AACTTTTAAAGCATACACAGTG	54.45		
DXS10079	AL049564	q12	66.632	90.82	AGAA	16–23	F-AGATTGTGCCAATGCTCTCCAG	55.18	133–191	
							R-AGGTATATTGGCCTGTAG	59.12		
DXS10135	AC003684	p22.31	9.199	20.03	GAAA	15–33	F-GCATTTGAAACTAAAGTCAAATG	58.19	201–313	
							R-AATACCTGGATCTAGCCAAGCCTG	55.51		
HPRTB	M26434	q26.2	133.443	149.66	AGAT	10–16	F-GTAGTTTCTTAAAGTGTCTC	53.15	323–375	
							R-ATACACATCCCCATTCCTG	54.85		

### Primer design

The reference sequence for each locus was obtained from GenBank (http://www.ncbi.nlm.nih.gov/genbank/). Primers were designed in Primer Premier 5.0 (PREMIER Bio-soft International, Palo Alto, CA), with 18–26 base pairs (bp) in length, Tm from 55 to 65 °C, and amplicon size from 86 to 404 bp (240 bp on average). Stringent criteria were also imposed to minimize primer dimers and hairpin structures, and to ensure uniqueness against the entire reference genome. Finally, potential interactions among primers were examined in AutoDimer v1.1^5^. One primer from each pair was fluorescently labeled (FAM, HEX ,TAMRA or ROX) at the 5′ end for capillary electrophoretic (CE) detection^6^.

### Amplification

DNA (1 ng) was amplified in a single 25 µL reaction containing 10 µL 2.5 × Buffer C (100 mM KCl, 1.5 mM MgCl_2_, 0.2 mM dNTPs, 50 mM Tris–HCl, 100 mM Betaine, 0.16 mg/mL BSA, 0.001 mM Brij58, 0.40% DMSO, and 20 mM DTT), 5 µL 5 × primer set, and 0.5 µL Taq DNA Polymerase II (Microread Genetics Incorporation, Suzhou, China). Reactions consisted of pre-denaturation at 96 °C for 2 min on a GeneAmp PCR System 9700 with a gold-plated silver block (Applied Biosystems, Foster City, CA, USA), followed by 29 cycles of denaturation at 94 °C for 5 s, and annealing and extension at 60 °C for 70 s. Reactions were then held at 60 °C for 30 min, and finally stored at 15 °C.

### Electrophoresis

PCR products and allelic ladders (1 μL) were mixed with 0.5 μL internal size standard and 8.5 μL deionized Hi-Di Formamide (Thermofisher Scientific, Waltham, MA, USA), denatured, separated, and visualized on an ABI 3130xl or Genetic Analyzer (Thermofisher Scientific, Waltham, MA, USA). Samples were injected at 3 kV for 10 s and electrophoresed at 15 kV for 1,591 s in Performance Optimized Polymer-4 (Thermofisher Scientific, Waltham, MA, USA). Data were analyzed in GeneMapper ID-X v1.2 (Life Technologies, CA, USA). Unless stated otherwise, allele peaks were called at greater than 50 relative fluorescence units.

To generate internal size markers, various fragments were amplified from nucleotides 927 to 1425 of the pUC18 plasmid (Takara, Dalian, China), using a universal forward primer labeled with Microreader Org500 Dye (Microread Genetics Incorporation, Suzhou, China), and a set of reverse primers to amplify 13 fragments with size 75, 100, 139, 150, 160, 200, 300, 340, 350, 400, 450, 490, and 500 bp^7^.

A combination of individual templates representing the range of alleles observed in populations was used to construct allelic ladders. Briefly, each locus was amplified from different samples, and amplification products were diluted, pooled, re-amplified, and optimized to produce an allelic ladder with comparable peak height. Allelic ladders at each locus were then pooled at appropriate ratios to generate a ‘cocktail’^8^. All alleles in the ladder were sequenced to confirm number of repeats. Microreader 5-Dye Matrix Standards (Microread Genetics Incorporation, Suzhou, China) were used to calibrate electrophoresis instruments^9^ .

### Assessment of PCR conditions

Since robust PCR reactions are important to obtain reliable genotypes, we evaluated the PCR conditions for the multiplex amplification including annealing temperatures (56, 58, 60, 62, and 64 °C), number of cycles (27–32), final extension times (0, 15, 30, 45, and 60 min), Taq polymerase concentrations (0, 0.1, 0.2, 0.5, 0.75, 1.0, and 1.25 U), as well as buffer and primers at ± 10%, ± 20%, and ± 30% of optimal concentrations (descripted in the amplification section). All tests were based on 1 ng F312 control DNA.

### Sensitivity and stability studies

To evaluate the minimum quantity of DNA required to obtain reliable results, 0.03125, 0.0625, 0.125, 0.25, 0.5, and 1 ng control DNA F312 was amplified in triplicate using optimal parameters (descripted in the amplification section). Mean peak height was determined for each reaction, along with percentage of successfully genotyped alleles^8^. Forensic samples are often containing PCR inhibitors. Thus, stability studies were also carried out by adding 1 ng F312 control and different concentrations of three common inhibitors in forensic sample : hematin (80, 100, 120, 150, 200, 300, and 400 μM), humic acid ( 20, 40, and 60 ng/μL), tannic acid ( 10, 15, 20, and 25 ng/μL).

### Allele size accuracy and stutter ratio

Allele size, as well as the difference from expected size, was evaluated over multiple electrophoresis runs on the 3130xl and 3500xl Genetic Analyzer, repectively. The percentage of observed stutter at 19 X-STR loci was assessed in 357 samples by calculating the ratio of the stutter peak height compared to the corresponding allele peak height^10^.

### Species specificity

Although this multiplex typing system is designed for human samples, the ability to detect genetic information from non-targeted species should be examined. Non-human DNA samples from common domestic animal species (10 ng DNA each from chicken, mouse, cat, dog, pig, sheep, and donkey) and 2 ng DNA from soil microbiota were amplified and electrophoresed as described, using 1 ng control DNA F312 as positive control.

### Concordance and reproducibility

A total of 184 bloodstain samples from unrelated individuals were analyzed with our multiplex system to detect the concordance and reproducibility.

### Casework samples

The ability to obtained accurate and reliable genotypes from casework samples could indicate if our multiplex system would be widely applied to the field of forensic science. A total of 35 samples, including 6 bloodstains, 6 saliva stains, 5 hair root samples, 7 muscle tissues, 6 old bones and 5 semen samples were examined by our multiplex system. Each casework sample had been tested twice independently. Genomic DNA from bloodstain, saliva stain, hair root, semen and muscle tissue samples were extracted using ﻿QIAamp DNA Investigator Kit (QIAGEN, Hilden, Germany) according to the manufacturer’s manuals. Bone samples were extracted using the Automate Express and the Prepfiler Express BTA Forensic DNA Extraction Kit. Negative (no template DNA) and reagent blank controls were included on each experiments. Using the casework samples in this study were approved by the Ethical Committee of Shanxi Medical University ( Issued Number: 2020GLL031). The Ethical Committee of Shanxi Medical University was also agreed to waive the need for informed consent for these samples. All samples were de-identified before genotyping.

### Mixture studies

In forensic cases, samples frequently consist of DNA from more than one individual. Therefore, it is essential to evaluate the performance of the typing system for DNA mixtures detection. Mixtures of female 9947A and male 9948 DNA samples were examined in duplicate at various ratios (1:0, 1:1, 3:1, 5:1, 8:1, and 0:1), with total amount of DNA constant at 1 ng.

### Statistical analysis

Allele genotypes for all 19 loci were determined in 1085 samples from Beijing Han Chinese. Calculation of allele frequencies, exact tests of Hardy–Weinberg equilibrium in female samples, and pairwise exact test of linkage disequilibrium were performed in Arlequin Ver 3.5^11^. Power of discrimination in males and females, mean exclusion chance for standard trios with daughters, and mean exclusion chance for father/daughter duos were calculated using the online tool of the Forensic ChrX Research database (http://www.chrx-str.org).

## Results and discussion

### Multiplex design

**Figure 1:**
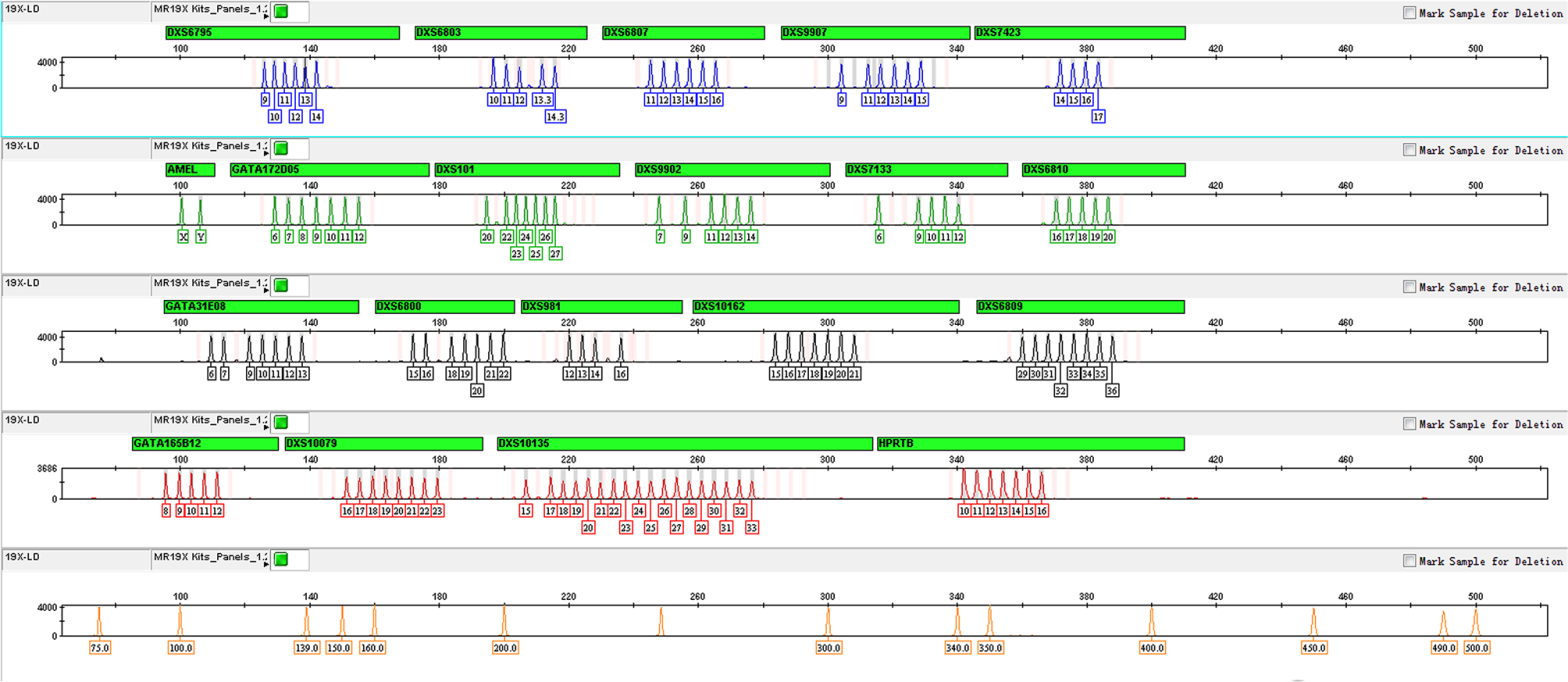
Electropherograms of allelic ladders and the internal size standards designed for the multiplex system. The four allelic ladders panels (from top to bottom) represent the FAM (blue), HEX (green), TAMRA (yellow), ROX (red) dye-labeled peaks with allele numbers underneath each peak. The fifth panel shows internal size standards labeled with Orange500 dye (75, 100, 139, 150, 160, 200, 250, 300, 340, 350, 400, 450, 490, and 500 bp).

A panel of multiplex-fluorescent-labeled typing system allows the detection of 19 X-STRs and amelogenin has been successfully developed. The final primers were designed to amplify short fragments, 240 bp on average, to increase the chances of successful amplification from degraded DNA, which is common in forensic samples. Primer sequences, melting temperatures, PCR product sizes, and dye labels are listed in Table 1. Allelic ladders and internal size markers are shown in Fig. 1, and genotypes of control DNA samples 9947A, 9948, and F312 are listed in Table S1.

### PCR conditions

DNA polymerase activity is the key factor of STR genotyping, because non-specific amplification occurs when the concentration is too high, but the amplification efficiency is low when the concentration is too low. The results showed that amplification efficiency was comparable at 0.5–1.25 U Taq polymerase, but was sharply lower at 0.2 U and undetectable at 0.1 U (Fig. S2). Thus, at least 0.5 U Taq polymerase should be used in routine testing.

Pipetting errors can also lead to the change of buffer and primer concentration, especially in very small-volume reactions. We found that full profiles were obtained over a range of buffer and primer concentrations, although changes in peak height were observed (Figs. S3, S4). These results indicated that our multiplex system is robust, and tolerates variability in the concentration of PCR components.

The optimal number of amplification cycles can ensure the best performance of PCR amplification.As expected, peak height increased along with number of cycles. However, non-specific allele peaks and differences in peak height among loci were also observed with increasing number of cycles (Fig. S5), and we found 29 cycles to be optimal.

The annealing temperature was measured in increments of 2 °C from 56 °C to 64 °C. Although allele dropout was not observed, abnormal peak shape was noted in some loci, such as in GATA172D05 at 56 °C and 58 °C, as well as in DXS6810 at 62 °C and 64 °C. Thus, the optimal profiles can be obtained when annealing temperature is 60 °C (Fig. S6).

Most thermostable DNA polymerase reactions have the characteristic of adding an "a" to the 3-terminal of PCR products^12^. Non-template addition results in one base pair longer than the actual target sequence. The final extension step in the PCR reaction allows the DNA polymerase add A to all double-stranded PCR products. Thus, final extension times of 0, 15, 30, 45, and 60 min were tesed at standard annealing temperature (60 °C), respectively. Results showed that when the final extension time was more than 30 min, normal electrophoretic peaks could be observed at all loci. (Fig. S7).

### Sensitivity

**Figure 2:**
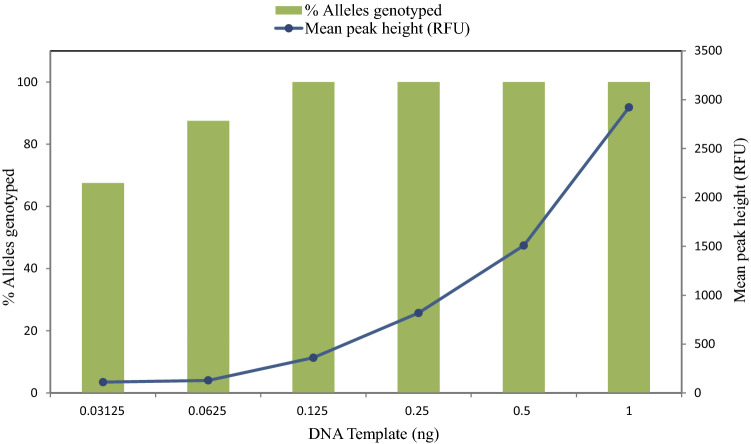
Sensitivity testing of control DNA F312 ranging from 1 ng to 31.25 pg. The left-hand y-axis indicates percentage of successfully genotyped alleles, while the right-hand y-axis indicates mean peak height.

Forensic samples often contain DNA below the recommended quantity. To determine the lowest amount of sample DNA from which a full profile (34 alleles for F312 control DNA) can be generated reproducibly, we amplified a dilution series of F312 DNA, setting peak height 50 relative fluorescence units as limit of detection, and a peak height ratio > 60% for allele at a heterozygous locus (Fig. 2). We found that full and reliable profiles without obvious dropouts can be generated from 0.125 ng template DNA, although peak height sharply decreased in comparison to 1 ng DNA. Dropout of GATA172D05 and GATA165B12 was observed at 0.0625 ng, along with variable peak height in DXS10079 and DXS10135. These four loci were not amplified at 0.03125 ng, at which GATA165B12 was also dropped, and peak height was variable in DXS10162, DXS6809, and HPRTB (Fig. S8). Thus, the lowest amount of DNA template for this multiplex was determined to be 0.125 ng.

### Allele size accuracy

**Figure 3:**
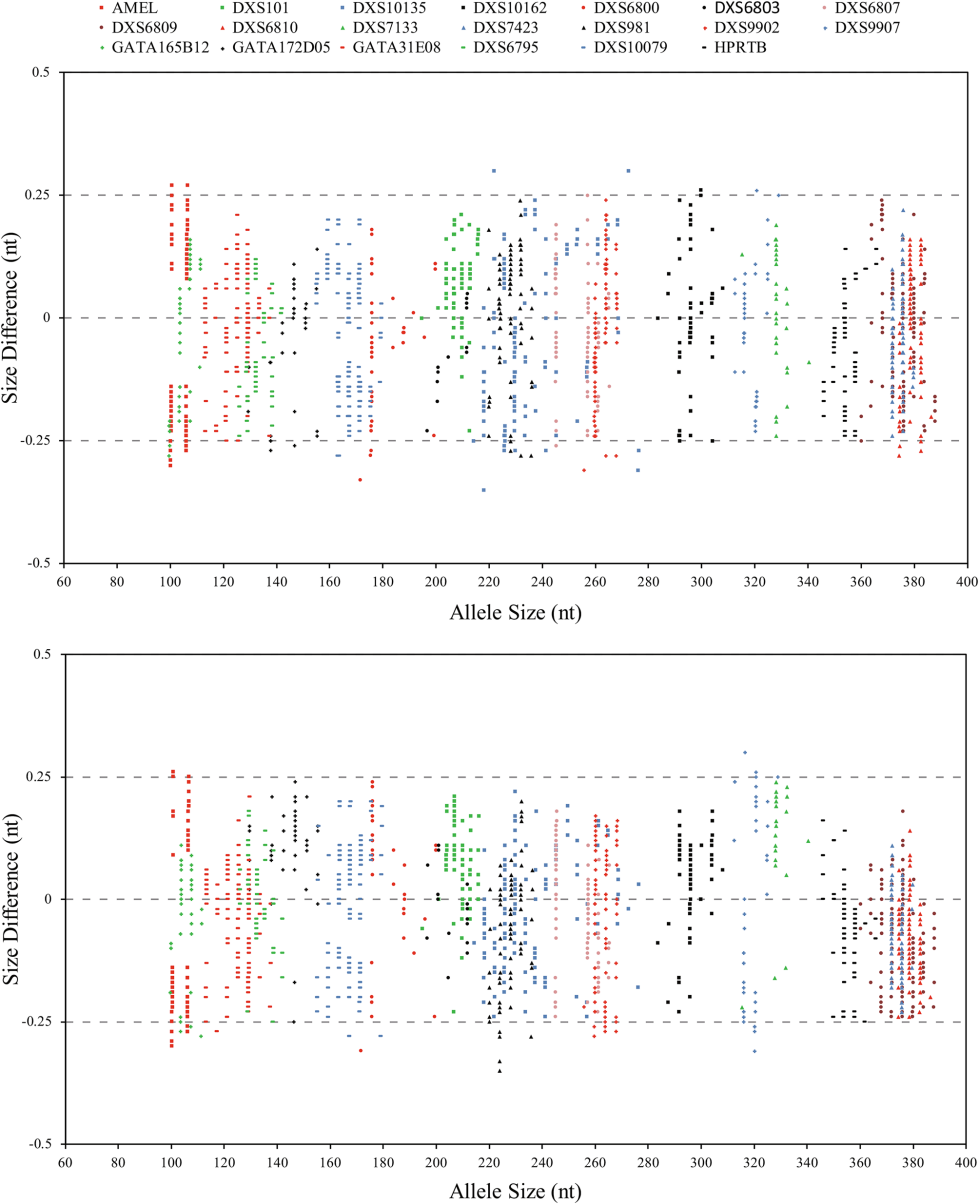
Accuracy of allelic ladders analyzed on a 3500xl Genetic Analyzer (n = 48, top) and on a 3130xl Genetic Analyzer (n = 32, bottom). The x-axis indicates allele size, while the y-axis represents difference from expected size.

Allele size accuracy allows for obtaining reliable and accurate genotypes. Size variations could be found between different CE instruments or even different runs on the same instruments. Currently, 3130xl and 3500xl Genetic Analyzers were the most commonly used CE instruments in forensic laboratory. All sample alleles are within ± 0.5 nucleotides from a corresponding allele in the Allelic Ladder, irrespective of the capillary electrophoresis platforms (Fig. 3). Thus, the recommended method for detection and correct assignment of alleles is to employ a ± 0.5-nt “window” around the size obtained for each allele in our multiplex typing system.

### Stutter ratio

Due to strand slippage along core repeat motifs during PCR, product that one repeat unit maller (or less frequently, one repeat larger) than the major STR product, so-called stutters, could be amplified^13–15^. The stutter ratio mean and standard deviations for each locus calculated from a subset of 357 samples are listed in Table 2. Of the 19 X-STR loci, the stutter ratio was lowest in DXS6800 (1.84%) and highest in DXS6809 (11.14%). The average stutter plus three standard deviations were used to set the stutter filter threshold, which could be used to determine whether the small peak was a stutter or minor component concealed in a mixture^16^. The stutter ratios for the studied loci were all below the stutter filter threshold, except for DXS6800, DXS6807, DXS6803, DXS10162, and DXS6809.

**Table 2:** % Stutter in 19 loci from 357 samples.

Marker	Mean	Stdev	Min	Max	Recommended filter
DXS6800	1.84	0.72	0.82	6.98	4.01
DXS7133	3.84	0.91	1.36	5.64	6.57
GATA172D05	4.12	1.08	2.27	5.76	7.36
DXS7423	4.37	0.71	3.01	6.15	6.51
GATA165B12	4.56	1.40	2.20	6.95	8.75
DXS6810	4.60	1.22	2.77	7.69	8.27
DXS6807	4.76	2.29	1.83	14.38	11.61
GATA31E08	4.80	1.57	2.02	7.49	9.51
DXS9902	4.95	1.00	3.27	7.18	7.94
DXS6803	5.08	0.89	3.30	8.33	7.75
DXS9907	5.16	1.09	3.73	7.01	8.43
DXS10162	6.38	0.87	4.50	9.21	8.99
DXS6795	6.58	2.72	1.11	10.93	14.74
DXS10079	6.67	1.30	3.02	9.03	10.58
DXS981	7.29	1.32	5.03	9.97	11.24
HPRTB	8.20	1.46	5.45	10.95	12.56
DXS10135	9.40	2.39	5.51	12.80	16.58
DXS101	11.12	2.10	1.88	16.06	17.43
DXS6809	11.14	1.60	7.38	18.46	15.94

### Stability

Since forensic samples are complex and often containing inhibitors, three common PCR inhibitors (i.e. humic acid, tannic acid, and hematin) were tested using the 19 X-STRs multiplex system. Results demonstrated this multiplex system could tolerate considerable concentrations of inhibitors. Full profiles from 1 ng F312 DNA were obtained in the presence of humic acid, tannic acid, and hematin with the concentration no greater than 20 ng/μL, 15 ng/μL, and 200 μM, respectively (Figs. S9–S11). Allele dropouts were observed at higher concentrations and the locus entirely dropped out when the concentration of humic acid, tannic acid, and hematin were further increased to 60 ng/μL, 25 ng/μL, and 400 μM. With the increased concentration of PCR inhibitors, the peak heights of all loci were also decreased and no profiles were obtained when the concentration of humic acid, tannic acid, and hematin were further increased to 60 ng/μL, 25 ng/μL, and 400 μM.

### Species specificity

When DNA templates of common domestic animal species (chicken, mouse, cat, dog, pig, sheep, and donkey) and microbial pool were used, no allele peak was observed at all loci (Fig. S12). This results demonstrated that the 19 X-STRs multiplex system is efficient for human-specific DNA testing.

### Concordance and reproducibility

The complete STR profiles were successfully obtained from all samples. The fluorescence signals of different loci were well-distributed with low noise ratio, smoother baseline and no interference peaks such as spectral penetration.

### Casework samples

The results showed that the complete STR genotypes were obtained from 35 samples without any allele dropout and all STR genotypes of were consistent between those two independent testing.

### DNA mixtures

**Figure 4:**
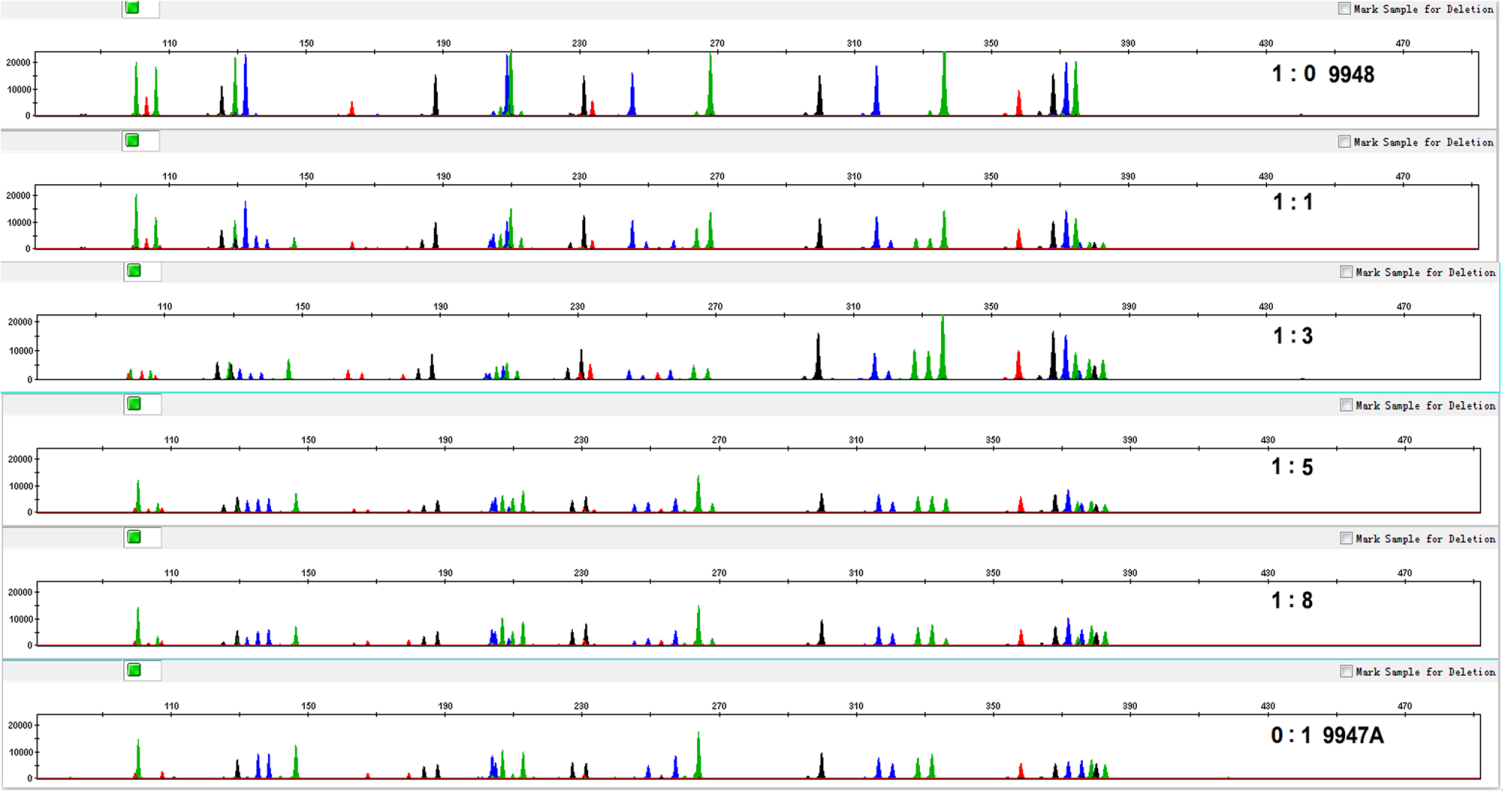
Representative electropherograms of a 1:0, 1:1, 1:3, 1:5, 1:8, and 0:1 mixture of control DNA 9948 and 9947A with a total of 1 ng of DNA.

Forensic samples often contain mixtures of DNA. Therefore, we mixed control DNA 9948 (male) and 9947A (female) at 1:0, 1:1, 1:3, 1:5, 1:8, and 0:1, keeping the total template DNA constant at 1 ng. No allele dropout was observed even at 1:8 ratio, although peak height was proportional to the amount of DNA (Fig. 4). This result suggested that the 19 X-STRs multiplex system was suitable to genotype samples with DNA from two individuals.

### Population studies and forensic parameters

A cohort of 1085 unrelated healthy Beijing Han individuals, 494 males and 591 females, were genotyped. Allelic frequency and forensic parameters for each X-STR locus are listed in Table 3. No significant deviation from Hardy–Weinberg equilibrium was observed for any of the loci after Bonferroni correction (*p* = 0.0026). Similarly, no detectable evidence of linkage disequilibrium was noted for the same pair of markers after Bonferroni correction (*p* = 0.0001) (Table S2. A total of 189 alleles were observed in 19 X-STR loci. More than 27 alleles were detected for DXS10135, which had the highest heterozygosity (0.9201), polymorphism information content (0.9146), power of discrimination in males (0.9201) and females (0.9881), and mean exclusion chance for standard trios with daughters (0.9146) and for father/daughter duos (0.8481). This locus was also the most polymorphic X-STR in five population samples from the Middle East (Albania, Iraq, Lithuania, Slovenia, and Turkey)^17^, Chinese Han from Hebei^18^, Guanzhong^19^ and Zhejiang^20^. Among these loci, DXS6800 was the least informative locus, which heterozygosity was 0.3254. This result was quite different with other populations, such as Austria (0.7322)^21^, Germany(0.7314)^22^ and Argentinean (0.6340)^23^. However, populations from Asia also showed lower heterozygosity at this locus, such as Japanese (0.2660)^24^, Korean (0.2513)^25^ and Shandong Han (0.3470)^26^. This suggested us that more population genetic data are required to optimize the loci selection for forensic purpose.

**Table 3:** Allele frequency of 19 X-STR loci and forensic parameters based on 985 unrelated individuals.

Allele	DXS10079	DXS101	DXS10135	DXS10162	DXS6795	DXS6800	DXS6803	DXS6807	DXS6809	DXS6810	DXS7133	DXS7423	DXS981	DXS9902	DXS9907	GATA165B12	GATA172D05	GATA31E08	HPRTB
6											0.0016						0.0654	0.0024	
7														0.0056		0.0008	0.0040	0.0869	
8											0.0048			0.0016	0.0016	0.0016	0.1427	0.0359	
9					0.0542						0.7297		0.0016	0.0159	0.0048	0.2321	0.1132	0.1914	
9.3							0.0016												
10					0.1826		0.0136				0.2121		0.0032	0.4745	0.0008	0.5694	0.4219	0.2089	0.0008
10.3							0.0008												
11					0.2344	0.0016	0.1595	0.3947		0.0008	0.0478			0.2982	0.0287	0.1659	0.2065	0.3740	0.0670
11.3							0.0877						0.0008		0.0024				
12					0.0478		0.1132	0.0152			0.0040		0.0303	0.1898	0.4139	0.0279	0.0463	0.0941	0.2624
12.1														0.0008					
12.3							0.4761						0.0933						
13					0.4402		0.0614	0.0263				0.0072	0.1491	0.0128	0.4569	0.0024		0.0064	0.3844
13.3							0.0766						0.1866						
14	0.0016		0.0024		0.0359		0.0048	0.3517		0.0016		0.3062	0.2783	0.0008	0.0758				0.2129
14.3							0.0016						0.0582						
15	0.0024		0.0008	0.0040	0.0040		0.0032	0.1834				0.6316	0.1348		0.0136				0.0630
15.3													0.0064						
16	0.0128		0.0016	0.0383	0.0008	0.8134		0.0271		0.0088		0.0478	0.0518		0.0016				0.0096
16.3													0.0040						
17	0.0478		0.0120	0.1691		0.0008		0.0016		0.1571		0.0072	0.0016						
17.2				0.0064															
18	0.0941	0.0008	0.0502	0.3557		0.0128				0.5598									
18.2	0.0016																		
19	0.2552	0.0032	0.1085	0.2703		0.0997				0.2632									
19.2	0.0008																		
20	0.2552	0.0008	0.1077	0.1196		0.0048				0.0088									
21	0.2185	0.0088	0.1284	0.0327		0.0159													
21.1			0.0008																
21.3			0.0008																
22	0.0797	0.0327	0.1164	0.0032		0.0510													
23	0.0263	0.1180	0.0853																
24	0.0040	0.2624	0.0710																
25		0.2456	0.0598																
26		0.1970	0.0415																
27		0.0702	0.0351																
28		0.0343	0.0391																
29		0.0199	0.0335						0.0016										
30		0.0024	0.0327						0.0199										
31		0.0040	0.0255						0.1411										
31.2			0.0008																
32			0.0207						0.1786										
32.3				0.0008															
33			0.0096						0.2767										
34			0.0056						0.2193										
35			0.0040						0.1093										
36			0.0032						0.0431										
37			0.0032						0.0072										
38									0.0032										
He	0.8036	0.8104	0.9201	0.7549	0.7114	0.3254	0.7175	0.6852	0.8093	0.5925	0.4203	0.5049	0.8316	0.6494	0.6131	0.5937	0.7398	0.7621	0.7295
PIC	0.7760	0.7846	0.9146	0.7172	0.6698	0.3082	0.6901	0.6268	0.7826	0.5301	0.3697	0.4278	0.8113	0.5864	0.5364	0.5373	0.7061	0.7284	0.6851
PDf	0.9338	0.9382	0.9881	0.9023	0.8751	0.5277	0.8927	0.8425	0.9370	0.7715	0.6133	0.6778	0.9514	0.8141	0.7736	0.7785	0.8986	0.9098	0.8824
PDm	0.8036	0.8104	0.9201	0.7549	0.7114	0.3254	0.7175	0.6852	0.8093	0.5925	0.4203	0.5049	0.8316	0.6494	0.6131	0.5937	0.7398	0.7621	0.7295
MECI	0.7760	0.7846	0.9146	0.7172	0.6698	0.3082	0.6901	0.6268	0.7826	0.5301	0.3697	0.4278	0.8113	0.5864	0.5364	0.5373	0.7061	0.7284	0.6851
MECII	0.6524	0.6635	0.8481	0.5812	0.5274	0.1901	0.5499	0.4816	0.6601	0.3826	0.2387	0.2906	0.6981	0.4391	0.3930	0.3890	0.5681	0.5942	0.5441
HWE	0.7963	0.0336	0.6709	0.3636	0.7465	0.9301	0.3056	0.2415	0.1538	0.4375	0.1600	0.9343	0.3053	0.6158	0.0866	0.5258	0.3478	0.3786	0.2297

He, expect heterozygosity;PIC, polymorphism information content; PD_f_ ,power of discrimination in females; PD_m_, power of discrimination in males; MECI , mean exclusion chance for X-STR in standard trios with daughters;MECII , mean exclusion chance for X-STR in father/daughter duos;HWE, P-values for Hardy–Weinberg equilibrium.

The combined power of discrimination in females and males, as well as the combined mean exclusion chance in trios and duos, were 0.999999999999999995, 0.99999999995, 0.9999999995, and 0.9999996, respectively. All loci except DXS6800 showed high forensic efficiency values for the Beijing Han sample, which supports the application of the multiplex for forensic analyses.

## Conclusion

In this study, we intended to develop a sensitive multiplex X-STRs kit for forensic laboratories. The developmental validation studies here in tested the 19 X-STRs multiplex system following the SWGDAM Validation Guidelines. The testing results demonstrate that this typing system is robust and reliable for analyzing both forensic casework and database samples. Moreover, this multiplex typing system exhibited high forensic efficiency values for the Beijing Han samples.

## Supplementary Information

Supplementary Information.

## Data Availability

The data sets generated during the current study are available from the corresponding author on reasonable request.
