# Supplementary material for: Development and validation of a multiplex 19 X-chromosomal short tandem repeats typing system for forensic purposes
Source: Sci Rep. 2021 Jan 12;11:609. doi: 10.1038/s41598-020-80414-x (PMC7803774; doi:10.1038/s41598-020-80414-x)
Supplement: Supplementary file 1 — Supplementary Information. [file 41598_2020_80414_MOESM1_ESM.docx]

**Supplementary Information**

for

**Development and validation of a multiplex 19 X-chromosomal short tandem repeats typing system for forensic purposes**

Juan Jia ^1, *^, Xu Liu ^2, 3, *^, Qingwei Fan ^1, *^. Feng Cheng ^1^, Chen Fang ^2, 3^, Mengchun Wang ^1^, Jiarong Zhang ^1^, Wanting Li ^1^, Linyu Shi ^1^, Xiaomeng Zhang ^1^, Chuguang Chen ^4^.Zailiang Yu ^4^. Chen Li ^4^. Keming Yun ^1, #^, Jiangwei Yan ^1, #^

^1^ School of Forensic Medicine, Shanxi Medical University, Taiyuan 030001, China;

^2^ Beijing Center for Physical and Chemical Analysis, Beijing 10089, China

^3^ Beijing Engineering Technology Research Center of Gene Sequencing and Gene Function Analysis, Beijing 100089, China;

^4^ Beijing Microread Genetics Co., Ltd, Beijing 100044, China.

^*^ These authors contributed equally to this work.

^#^ Corresponding author:

Dr. Jiangwei Yan Dr. Keming Yun

School of Forensic Medicine, Shanxi Medicine University, Taiyuan 030001, Shanxi, China

Tel/Fax: +86-0351-3985377.

E-mail: [yanjw@sxmu.edu.cn](mailto:yanjw@sxmu.edu.cn), yunkeming5142@163.com


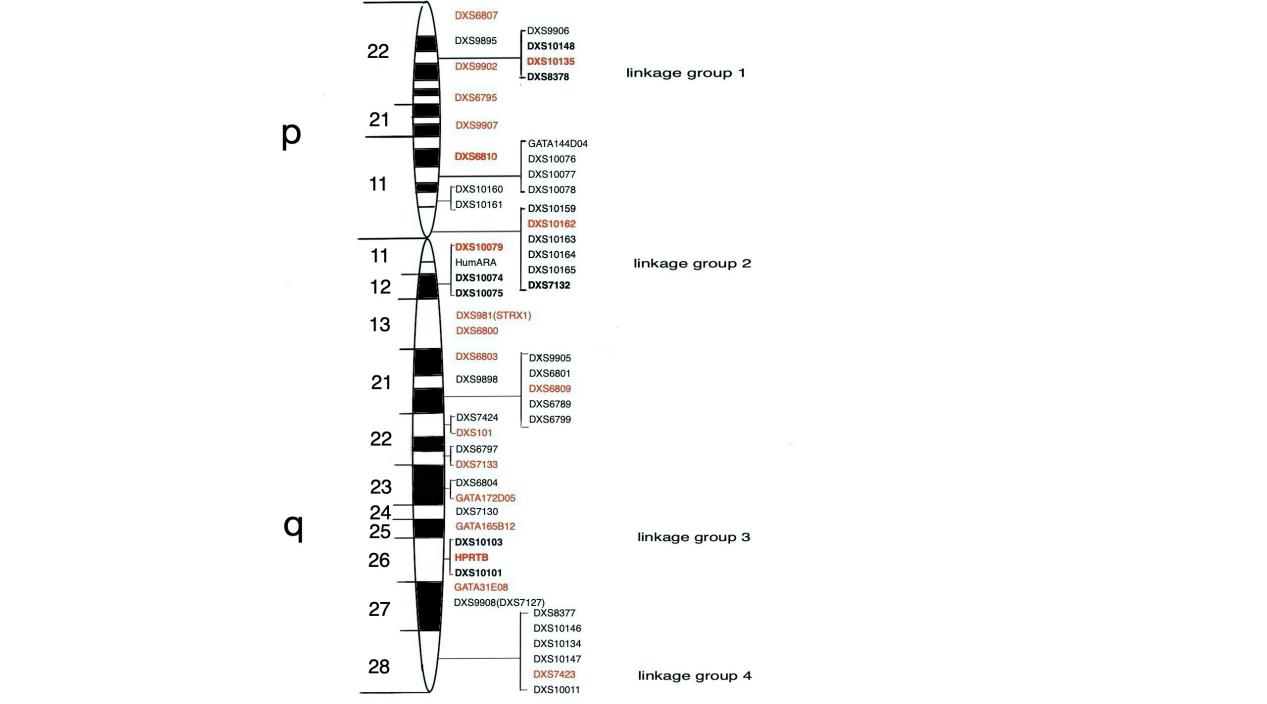


**Fig. S1.** Location of the 19 X-STR loci (boxed in red) used for the multiplex typing

system.


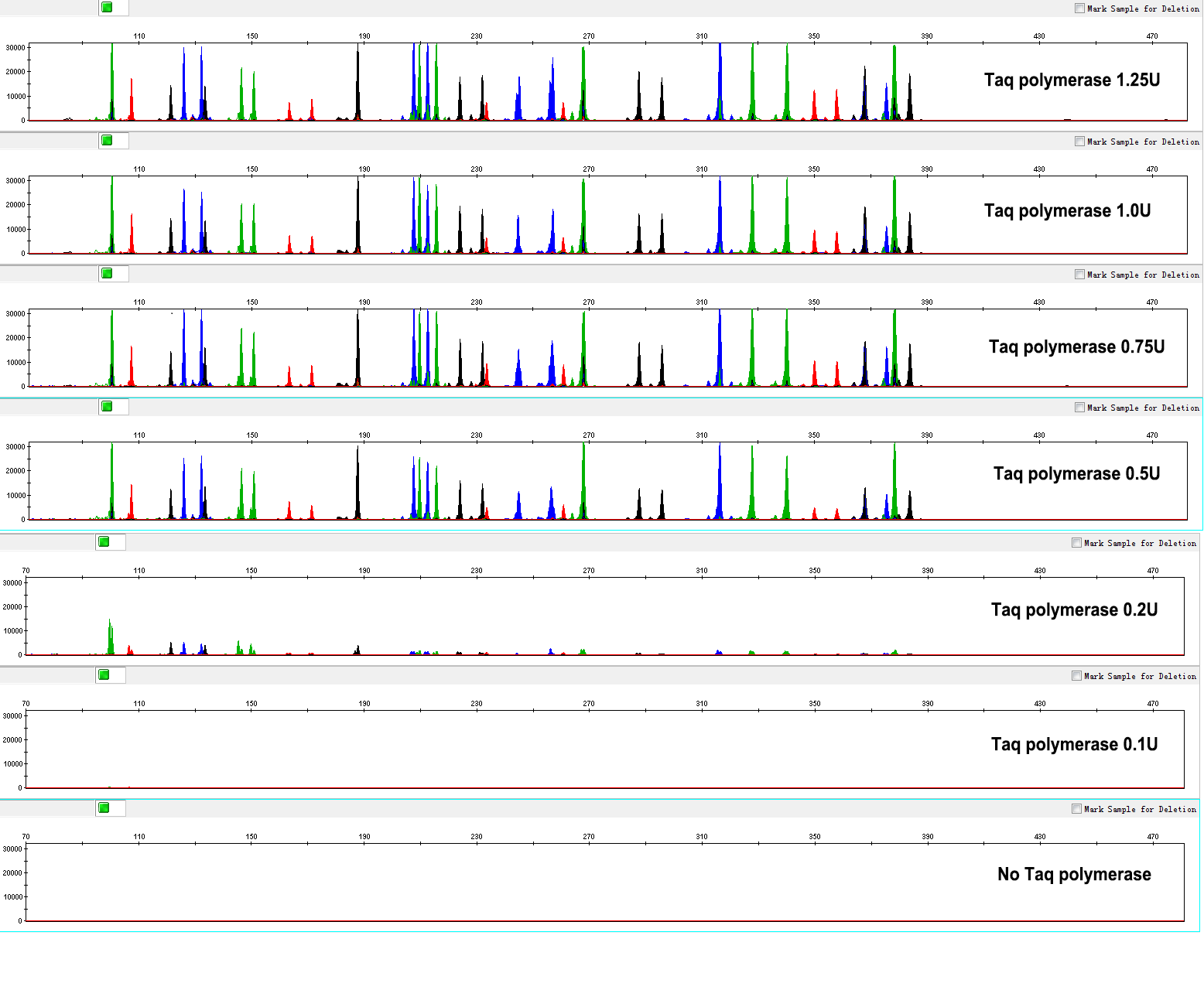


**Fig. S2.** Effect of various Taq polymerase concentrations on multiplex amplification. Seven concentration were tested : 1.25, 1.0, 0.75, 0.5, 0.2, 0.1 and 0 U (from top to bottom, respectively).


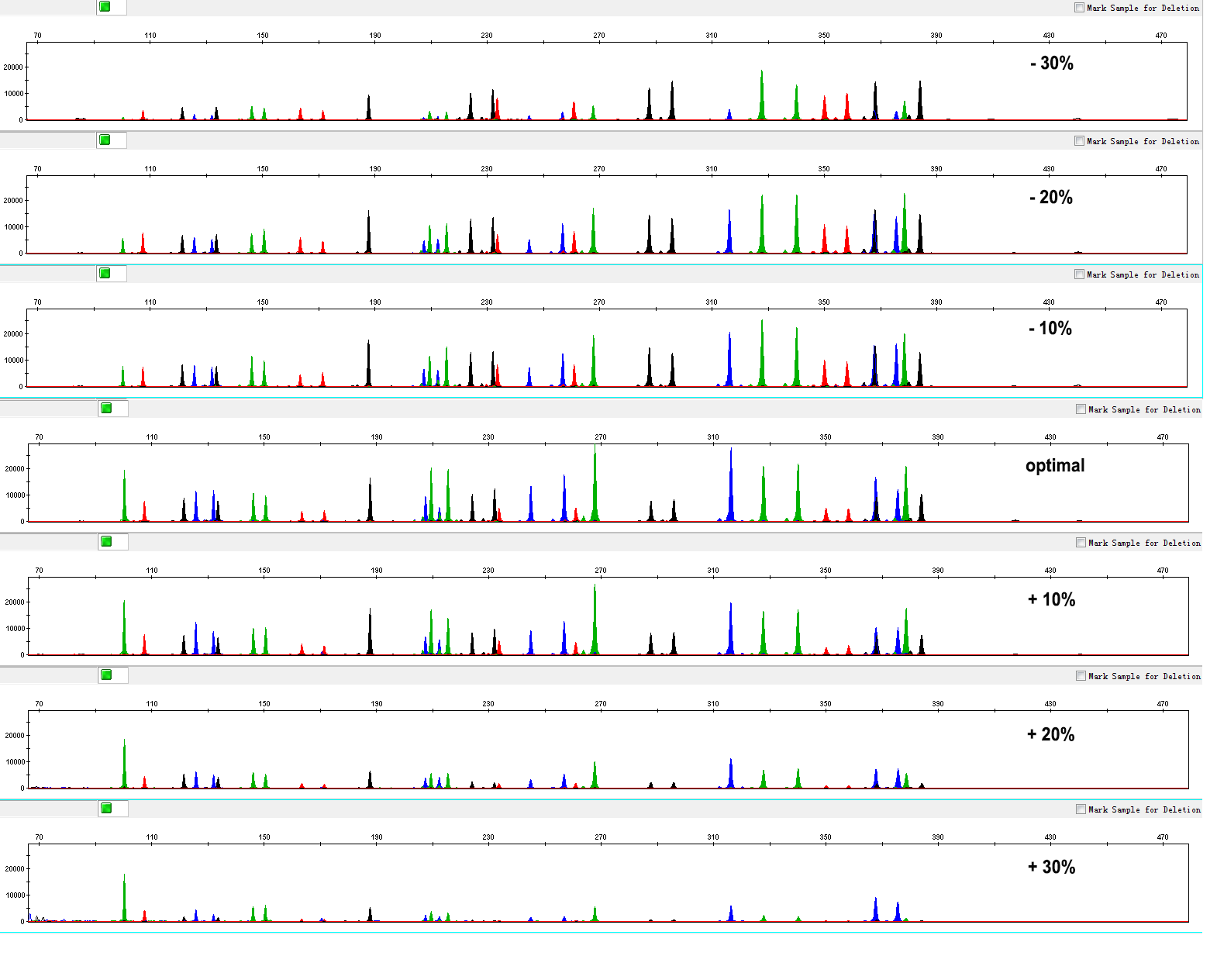


**Fig.** **S3.** Effect of various buffer concentration on multiplex amplification. Seven concentration were tested:± 30 %, ± 20 %, ±10 % of the optimal concentration.


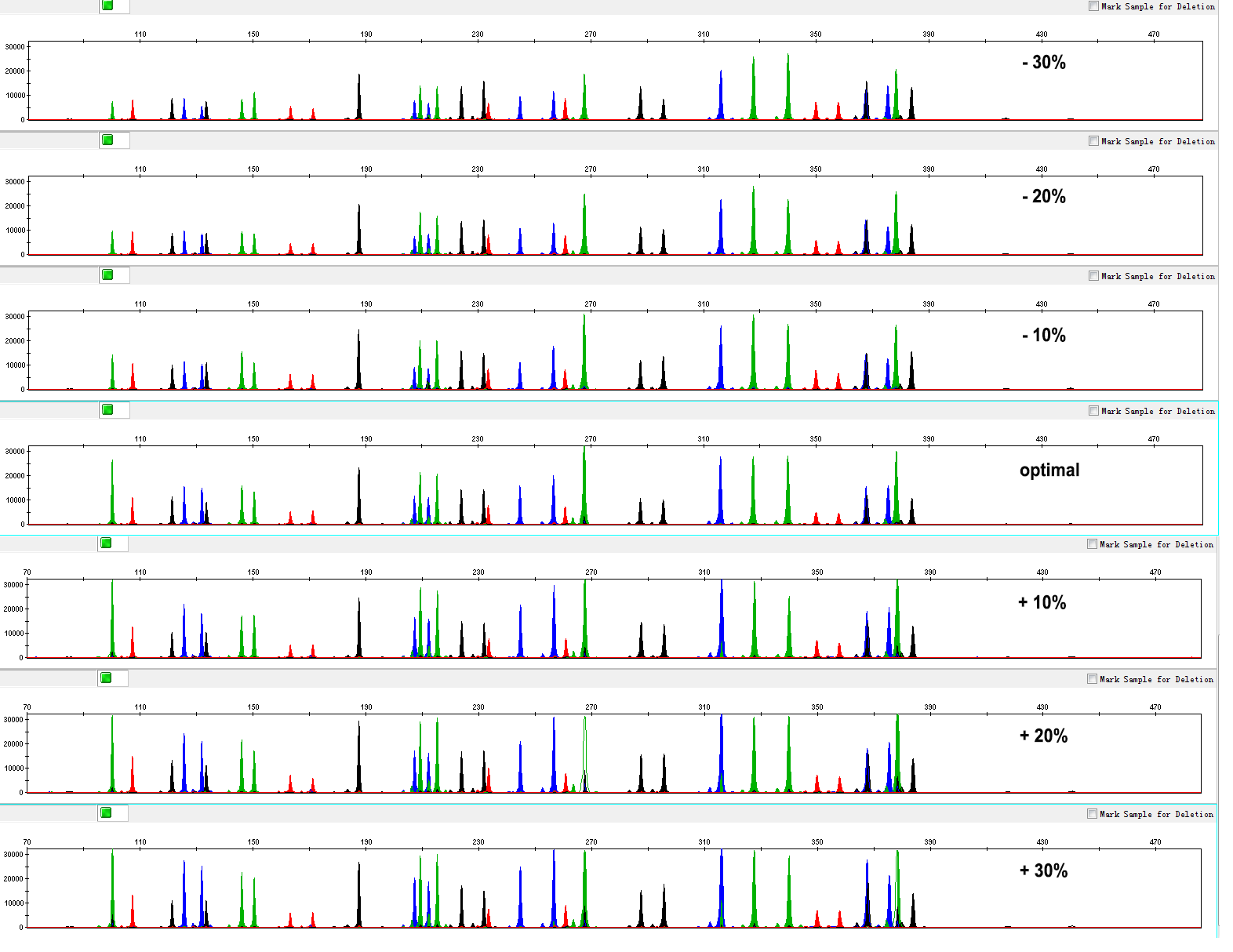


**Fig. S4.** Effect of various primer concentration on multiplex amplification. Seven concentration were tested:± 30 %, ± 20 %, ±10 % of the optimal concentration.


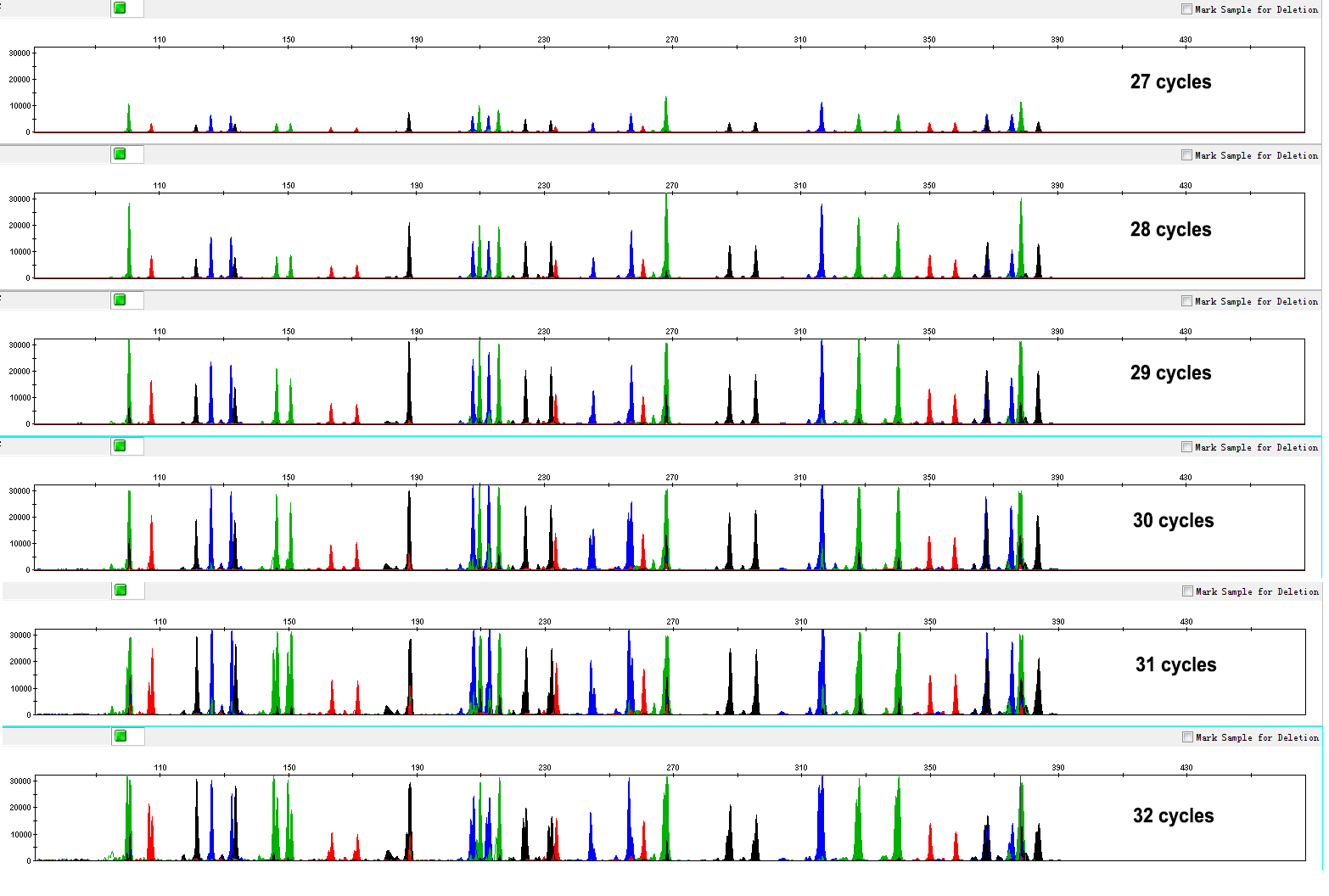


**Fig. S5.** Effect of various cycle numbers on multiplex amplification. Six amplification cycle numbers were tested: 27, 28, 29, 30, 31, and 32 (from top to bottom, respectively).


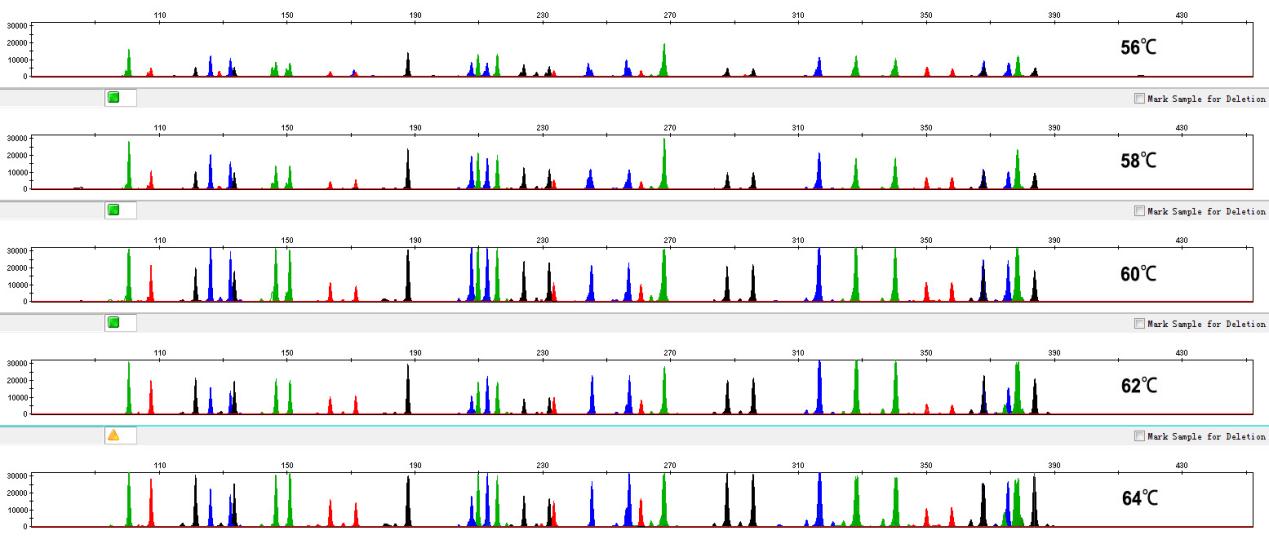


**Fig. S6.** Effect of various annealing temperatures on multiplex amplification. Five temperatures were tested: 56 °C, 58 °C, 60 °C, 62 °C, and 64 °C (from top to bottom, respectively).


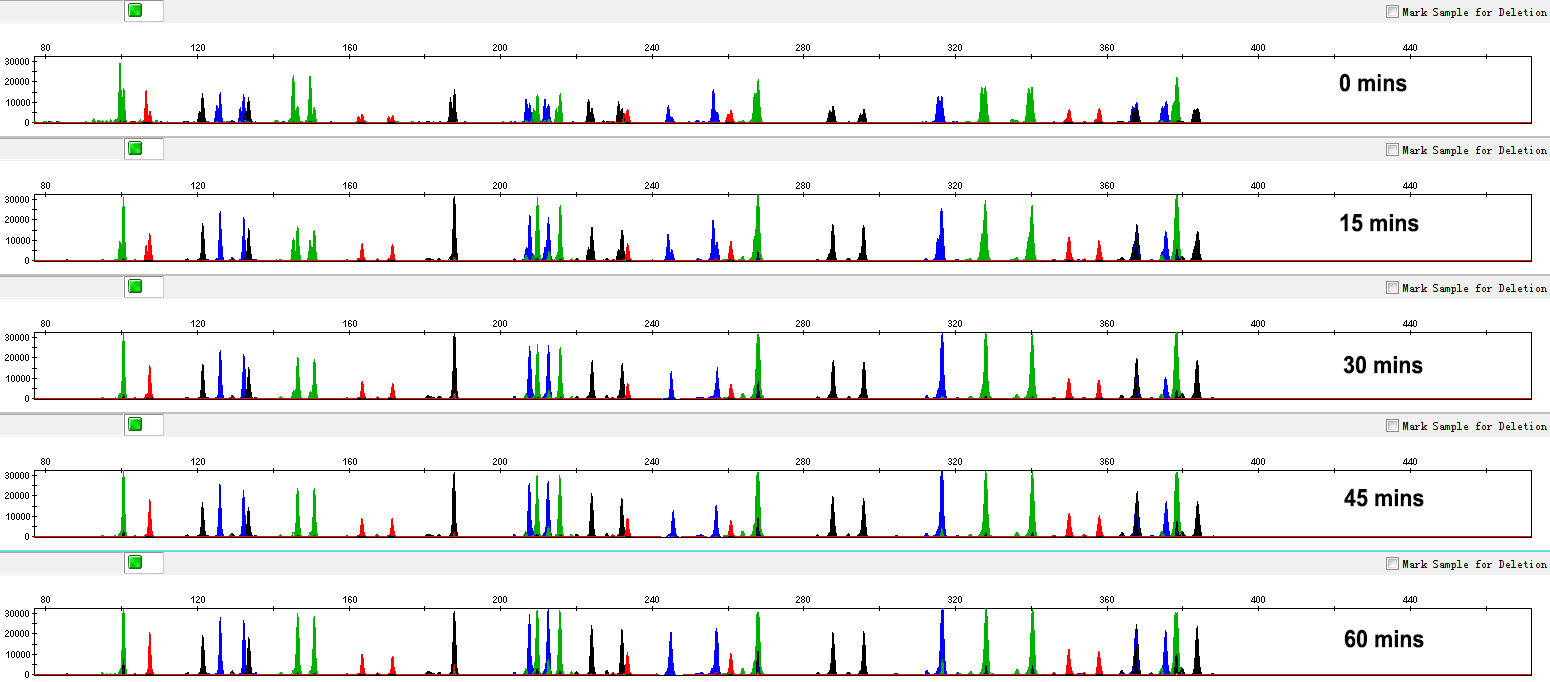


**Fig. S7.** Effect of various final extension time on multiplex amplification. Five times were tested: 0, 15, 30, 45, and 60 minutes (from top to bottom, respectively).


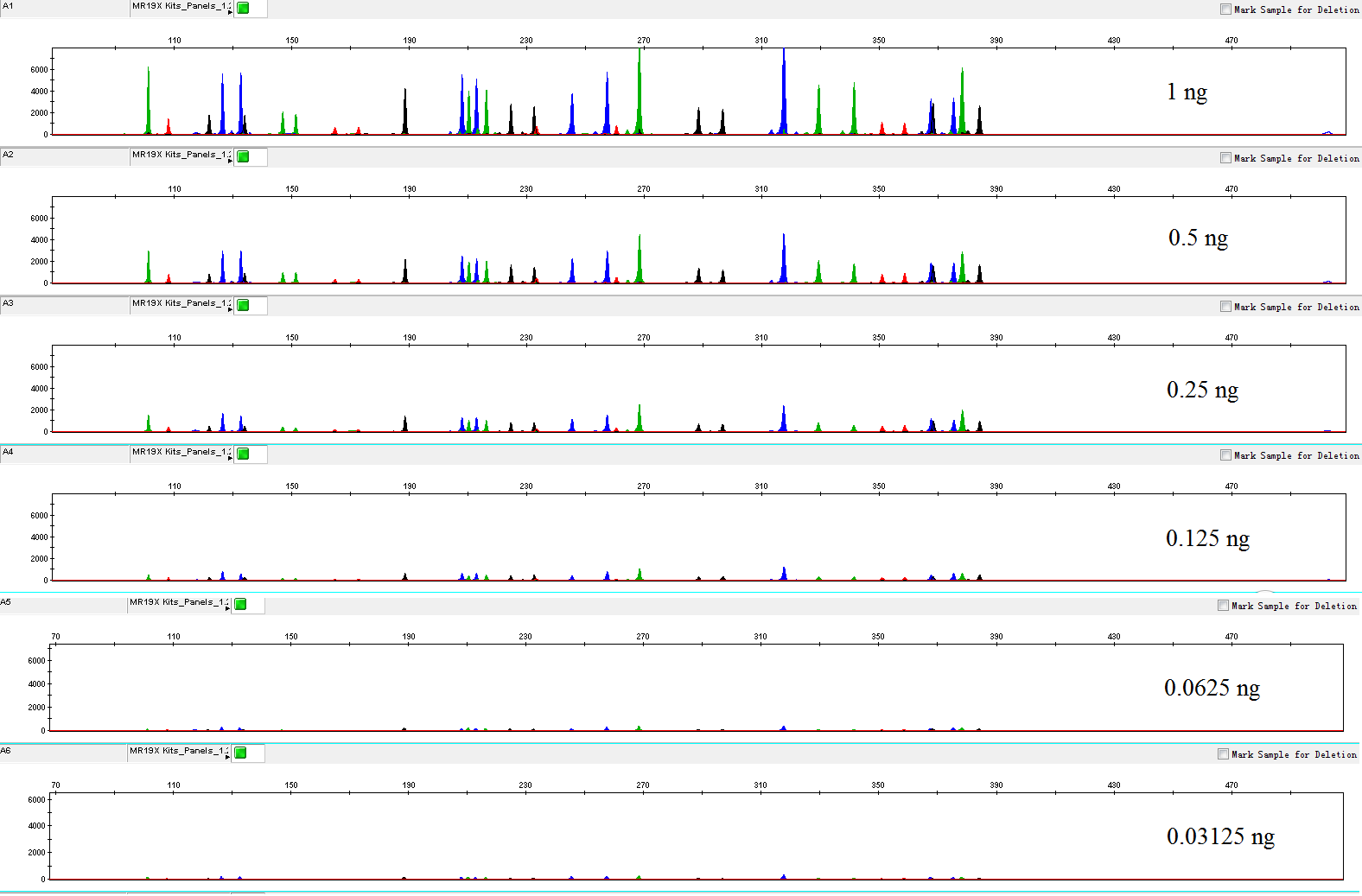


**Fig. S8.** Effect of various DNA template quantity on multiplex amplification. Six DNA template quantity were tested : 1, 0.5, 0.25, 0.125, 0.0625 and 0.03125 ng control DNA F312 was amplified (from top to bottom, respectively).


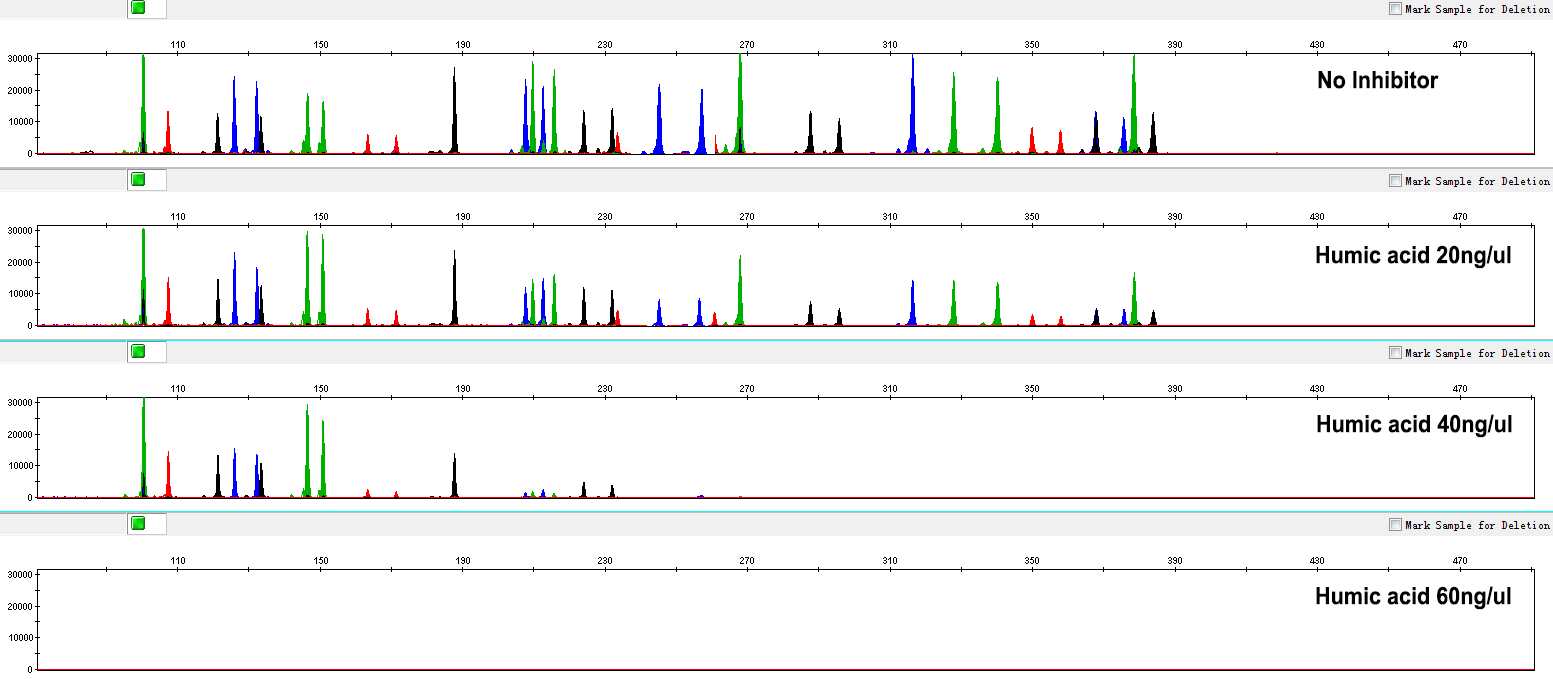


**Fig. S9.** Electropherograms obtained from amplification of a 1 ng F312 control DNA in the presence of various concentrations of humic acid (20, 40, and 60 ng/μL ).


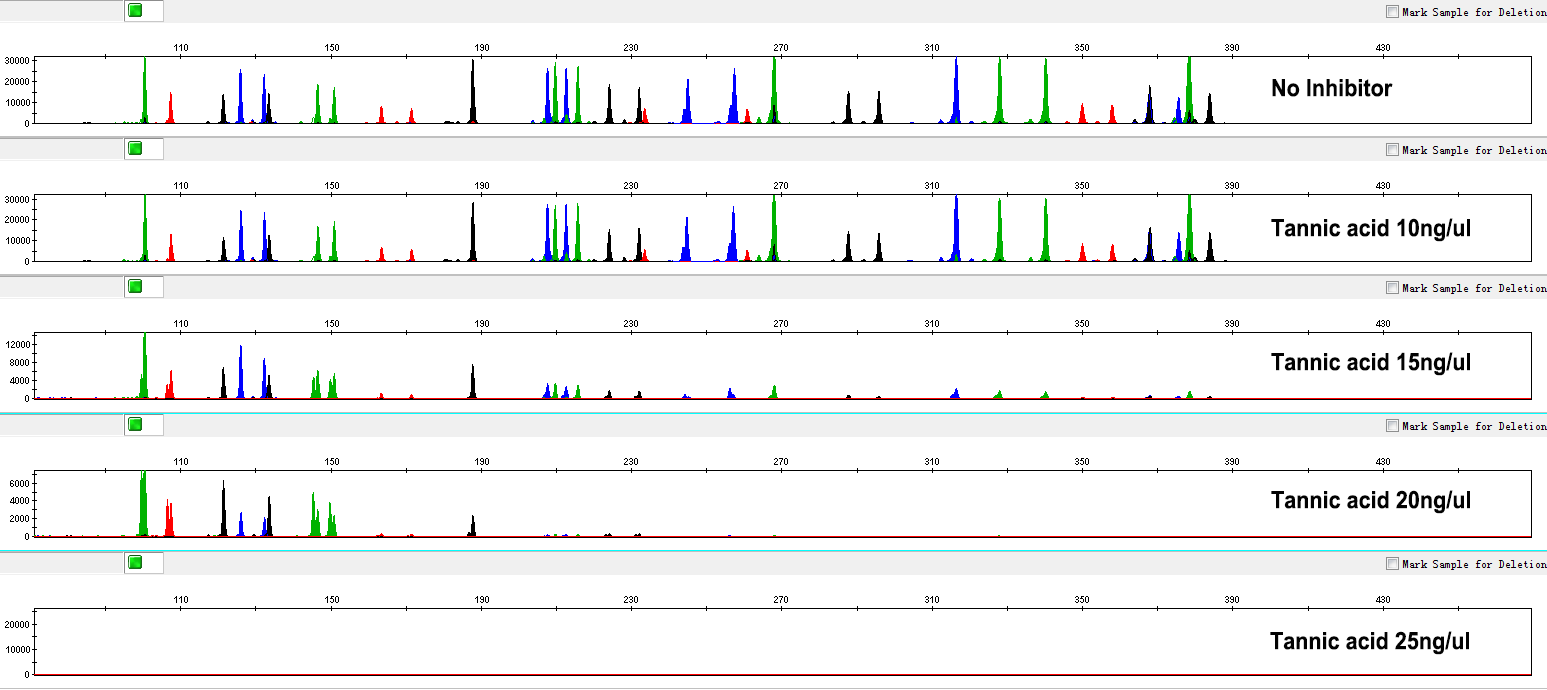


**Fig. S10.** Electropherograms obtained from amplification of a 1 ng F312 control DNA in the presence of various concentrations of tannic acid ( 10, 15, 20, and 25 ng/μL).


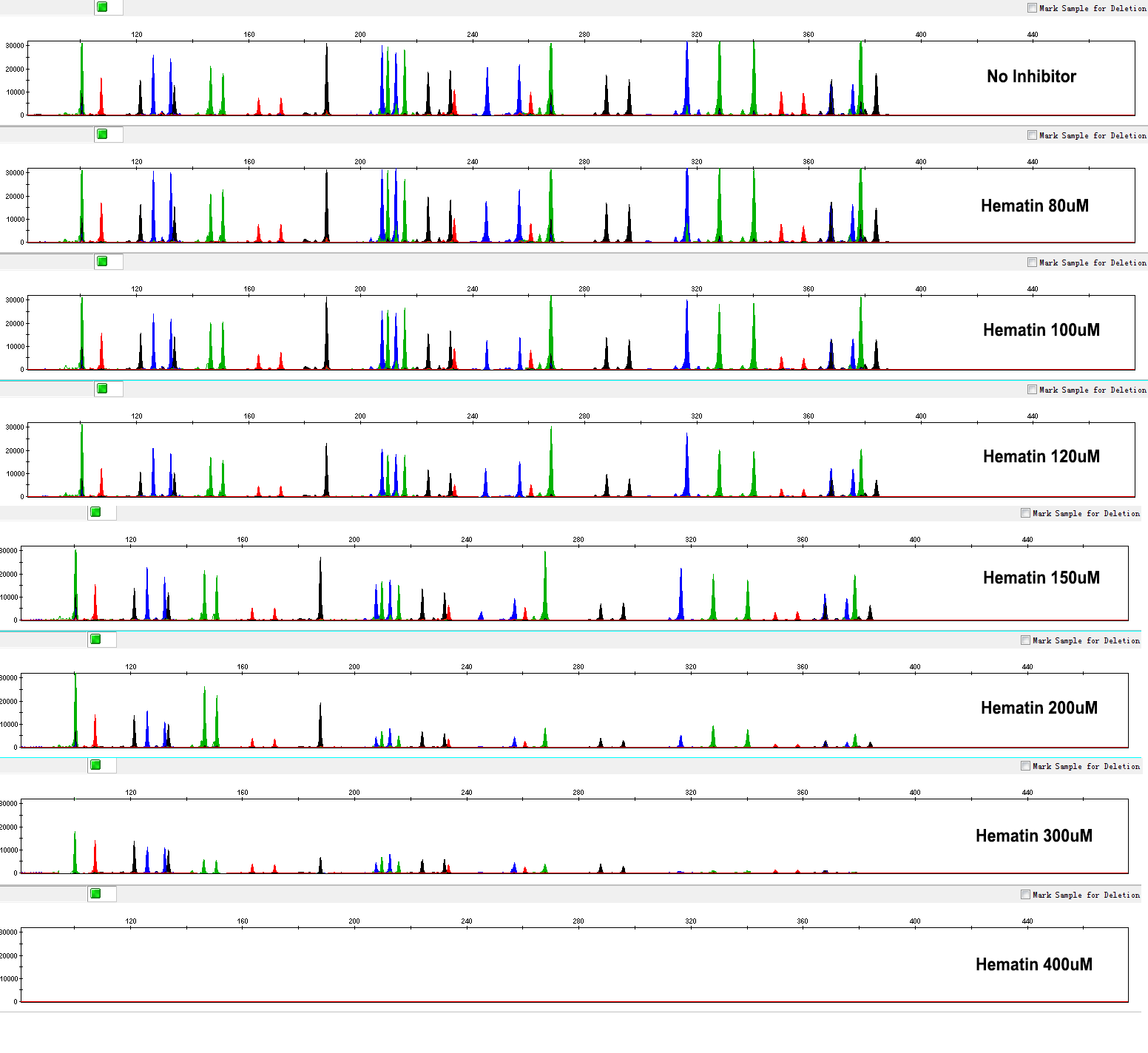


**Fig. S11.** Electropherograms obtained from amplification of a 1 ng F312 control DNA in the presence of various concentrations of hematin (80, 100, 120, 150, 200, 300, and 400 μM).


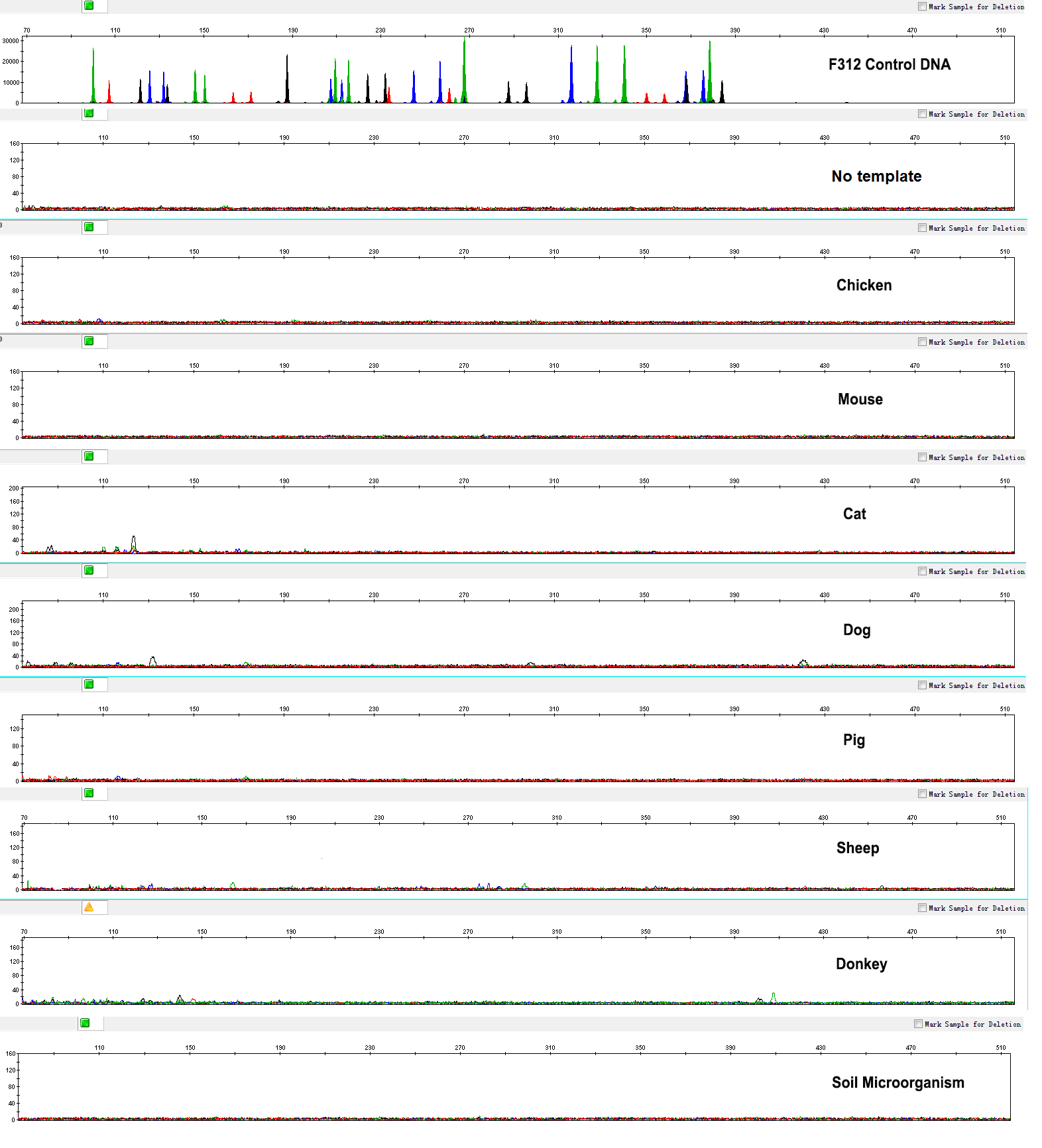


**Fig. S12.** Representative electropherograms for some species tested in a species specificity study.

| **Table S1.** Genotype of control DNA 9948, 9947A, and F312. | | | | | | |
| --- | --- | --- | --- | --- | --- | --- |
| Marker | 9948 | | 9947A | | F312 | |
|  | Allele 1 | Allele 2 | Allele 1 | Allele 2 | Allele 1 | Allele 2 |
| DXS6795 | 11 |  | 12 | 13 | 9 | 11 |
| DXS6803 | 13 |  | 11.3 | 12 | 12.3 | 14 |
| DXS6807 | 11 |  | 12 | 14 | 11 | 14 |
| DXS9907 | 12 |  | 12 | 13 | 12 | 12 |
| DXS7423 | 14 |  | 14 | 15 | 13 | 15 |
| AMEL | X | Y | X | X | X | X |
| GATA172D05 | 6 |  | 10 | 10 | 10 | 11 |
| DXS101 | 25 |  | 24 | 26 | 25 | 27 |
| DXS9902 | 12 |  | 11 | 11 | 12 | 12 |
| DXS7133 | 11 |  | 9 | 10 | 9 | 12 |
| DXS6810 | 17 |  | 18 | 19 | 18 | 18 |
| GATA31E08 | 10 |  | 11 | 11 | 9 | 12 |
| DXS6800 | 19 |  | 18 | 19 | 19 | 19 |
| DXS981 | 14.3 |  | 13.3 | 14.3 | 13 | 15 |
| DXS10162 | 19 |  | 19 | 19 | 16 | 18 |
| DXS6809 | 31 |  | 31 | 34 | 31 | 35 |
| GATA165B12 | 10 |  | 9 | 11 | 11 | 11 |
| DXS10079 | 19 |  | 20 | 23 | 19 | 21 |
| DXS10135 | 22 |  | 21.1 | 27 | 22 | 29 |
| HPRTB | 14 |  | 14 | 14 | 12 | 14 |

| **Table S2.** Linkage disequilibrium in 19 X-STRs in the Beijing Han. p values < 0.05 are indicated in bold. | | | | | | | | | | | | | | | | | | |
| --- | --- | --- | --- | --- | --- | --- | --- | --- | --- | --- | --- | --- | --- | --- | --- | --- | --- | --- |
| Locus | DXS10079 | DXS101 | DXS10135 | DXS10162 | DXS6795 | DXS6800 | DXS6803 | DXS6807 | DXS6809 | DXS6810 | DXS7133 | DXS7423 | DXS981 | DXS9902 | DXS9907 | GATA165B | GATA172D | GATA31E0 |
| DXS101 | **0.00434** |  |  |  |  |  |  |  |  |  |  |  |  |  |  |  |  |  |
| DXS10135 | 0.087784 | 0.474312 |  |  |  |  |  |  |  |  |  |  |  |  |  |  |  |  |
| DXS10162 | 0.201204 | 0.98201 | 0.46572 |  |  |  |  |  |  |  |  |  |  |  |  |  |  |  |
| DXS6795 | 0.893608 | 0.522624 | 0.30945 | 0.171604 |  |  |  |  |  |  |  |  |  |  |  |  |  |  |
| DXS6800 | 0.660042 | 0.255736 | 0.256038 | 0.401978 | 0.553734 |  |  |  |  |  |  |  |  |  |  |  |  |  |
| DXS6803 | 0.435594 | 0.158838 | 0.111418 | 0.88904 | 0.99799 | 0.238722 |  |  |  |  |  |  |  |  |  |  |  |  |
| DXS6807 | 0.337766 | 0.147148 | 0.063802 | 0.090482 | 0.372908 | 0.499568 | 0.273616 |  |  |  |  |  |  |  |  |  |  |  |
| DXS6809 | 0.056722 | 0.280726 | 0.963838 | 0.655924 | 0.096206 | 0.384204 | 0.089896 | 0.808046 |  |  |  |  |  |  |  |  |  |  |
| DXS6810 | 0.2612 | 0.154544 | 0.491392 | 0.285698 | **0.046626** | **0.030448** | 0.560282 | 0.729096 | 0.270676 |  |  |  |  |  |  |  |  |  |
| DXS7133 | 0.315582 | 0.864796 | 0.696334 | 0.924206 | 0.372566 | 0.65671 | 0.063828 | 0.579502 | 0.888608 | 0.781628 |  |  |  |  |  |  |  |  |
| DXS7423 | 0.107308 | 0.616726 | 0.994178 | 0.098256 | 0.947536 | 0.973188 | 0.550072 | 0.235212 | 0.619308 | 0.06741 | 0.487656 |  |  |  |  |  |  |  |
| DXS981 | 0.719196 | 0.332486 | 0.733042 | 0.803944 | 0.549624 | 0.137684 | **0.031072** | 0.270944 | 0.378386 | 0.48463 | 0.187424 | 0.712516 |  |  |  |  |  |  |
| DXS9902 | 0.68678 | 0.505256 | 0.911504 | 0.756644 | 0.900484 | 0.53149 | 0.171874 | 0.369988 | 0.34309 | 0.72157 | 0.285182 | 0.468724 | 0.076096 |  |  |  |  |  |
| DXS9907 | 0.60666 | 0.3623 | 0.978958 | 0.391306 | 0.73112 | 0.099456 | 0.50846 | 0.174884 | 0.830898 | 0.473948 | 0.84191 | 0.7718 | 0.488426 | 0.76816 |  |  |  |  |
| GATA165B | 0.603188 | 0.561612 | 0.91927 | 0.562806 | 0.583758 | 0.646018 | 0.391224 | 0.374002 | 0.365734 | 0.39836 | 0.31215 | 0.2673 | 0.540356 | 0.076314 | 0.165072 |  |  |  |
| GATA172D | 0.321562 | 0.822326 | 0.913228 | 0.561546 | 0.390138 | 0.919094 | 0.28641 | 0.272562 | 0.602092 | 0.59802 | 0.44892 | 0.860392 | 0.676046 | 0.358916 | 0.712692 | 0.279968 |  |  |
| GATA31E0 | 0.227404 | 0.342872 | 0.765086 | **0.01125** | 0.240632 | 0.335594 | 0.627454 | **0.012162** | 0.259242 | 0.150536 | 0.713242 | 0.666326 | 0.932172 | 0.898124 | 0.805654 | 0.41596 | 0.722974 |  |
| HPRTB | 0.639248 | 0.555214 | 0.557482 | **0.013606** | 0.91216 | 0.980898 | 0.798838 | 0.80237 | 0.381974 | 0.278104 | 0.45429 | 0.319766 | 0.13445 | 0.325266 | 0.855558 | 0.658392 | 0.50006 | 0.096162 |
